# Decoding of Ankle Flexion and Extension from Cortical Current Sources Estimated from Non-invasive Brain Activity Recording Methods

**DOI:** 10.3389/fnins.2017.00733

**Published:** 2018-01-08

**Authors:** Alejandra Mejia Tobar, Rikiya Hyoudou, Kahori Kita, Tatsuhiro Nakamura, Hiroyuki Kambara, Yousuke Ogata, Takashi Hanakawa, Yasuharu Koike, Natsue Yoshimura

**Affiliations:** ^1^Institute of Innovative Research, Tokyo Institute of Technology, Yokohama, Japan; ^2^Center for Frontier Medical Engineering, Chiba University, Chiba, Japan; ^3^Department of Advanced Neuroimaging, Integrative Brain Imaging Center, National Center of Neurology and Psychiatry, Tokyo, Japan

**Keywords:** brain computer interface, walking, electroencephalography, functional magnetic resonance imaging

## Abstract

The classification of ankle movements from non-invasive brain recordings can be applied to a brain-computer interface (BCI) to control exoskeletons, prosthesis, and functional electrical stimulators for the benefit of patients with walking impairments. In this research, ankle flexion and extension tasks at two force levels in both legs, were classified from cortical current sources estimated by a hierarchical variational Bayesian method, using electroencephalography (EEG) and functional magnetic resonance imaging (fMRI) recordings. The hierarchical prior for the current source estimation from EEG was obtained from activated brain areas and their intensities from an fMRI group (second-level) analysis. The fMRI group analysis was performed on regions of interest defined over the primary motor cortex, the supplementary motor area, and the somatosensory area, which are well-known to contribute to movement control. A sparse logistic regression method was applied for a nine-class classification (eight active tasks and a resting control task) obtaining a mean accuracy of 65.64% for time series of current sources, estimated from the EEG and the fMRI signals using a variational Bayesian method, and a mean accuracy of 22.19% for the classification of the pre-processed of EEG sensor signals, with a chance level of 11.11%. The higher classification accuracy of current sources, when compared to EEG classification accuracy, was attributed to the high number of sources and the different signal patterns obtained in the same vertex for different motor tasks. Since the inverse filter estimation for current sources can be done offline with the present method, the present method is applicable to real-time BCIs. Finally, due to the highly enhanced spatial distribution of current sources over the brain cortex, this method has the potential to identify activation patterns to design BCIs for the control of an affected limb in patients with stroke, or BCIs from motor imagery in patients with spinal cord injury.

## Introduction

Patients with walking impairments often use wheelchairs for transportation; however, this transportation means can cause pressure soars in the long-term if the patients do not perform pressure-relieving movements frequently (Stockton and Parker, 2002). Other devices such as exoskeletons and functional electrical stimulation (FES) systems allow patients to stand up and take steps however their control rely on the patient's unaffected motor abilities (pressing switches, trunk shifts, remaining muscle activity, etc.; Gancet et al., 2012; Contreras-Vidal et al., 2016). An alternative of control for these systems, which rely less heavily on the remaining motor abilities, is a brain-computer interface (BCI), in which brain signals are recorded and translated into control commands for assistive and communication devices (Wolpaw et al., 2002; Shih et al., 2012).

Breakthroughs in BCI have demonstrated the potential of this technology for motor rehabilitation, by controlling virtual environments and upper limb robots from implanted electrodes on the brain cortex (Wessberg et al., 2000; Serruya et al., 2002; Taylor et al., 2002; Carmena et al., 2003; Velliste et al., 2008; Hochberg et al., 2012). A study by Fitzsimmons et al. (2009) in non-human primates using implanted electrodes, demonstrated that it is possible to decode bipedal walking patterns (leg kinematics and EMG activities), from cortical ensembles in M1 and S1 during forward and backward walking tasks, showing the feasibility to use invasive BCIs for the restoration of gait in humans with intact locomotion centers in the brain. While invasive recordings can provide signals with high spatial resolution that allow for the decoding of more kinematic and physiological variables relevant to gait, the need for surgery limits the population that can access to this technology and, furthermore, there is always a risk of infection with implanted electrodes (Lesser et al., 2010). In this sense, non-invasive BCI techniques are preferred because they are safer and patients do not need to meet strict inclusion criteria to participate in this type of BCI studies. Among non-invasive techniques, electroencephalography (EEG) has a high temporal resolution and therefore is suitable for real time applications; nevertheless, movement artifacts and other sources of noise easily affect it. Despite its limitations, EEG is widely used in BCIs because of its portability.

Studies of BCIs based on binary classifications (i.e., detection of movement intention) using EEG have shown promising results for the development of assistive devices for gait restoration in patients with movement impairments (Waldert et al., 2008; King et al., 2013; Xu et al., 2014; Barsotti et al., 2015; Jiang et al., 2015; Severens et al., 2015; Yang et al., 2015; Pereira et al., 2017). Furthermore, long-term training with non-invasive BCIs has showed significant improvement in cortical plasticity in M1 and S1 areas in patients with paraplegia (Donati et al., 2016), demonstrating potential in the practical use of BCIs for the rehabilitation of walking impairments. However, in order to design a more natural non-invasive BCI for walking, it is necessary to classify more categories of motor tasks. Since the motor areas in the brain that represent right and left leg and foot are in close proximity (Meier et al., 2008), the multi-class classification of motor tasks in the lower limbs becomes a more challenging task for EEG signals.

To improve the spatial resolution and classification accuracies, various techniques to estimate cortical current sources from EEG, magnetoencephalography (MEG) and functional magnetic resonance imaging (fMRI) have been developed (Baillet et al., 2001). Among these techniques, we use a hierarchical Bayesian method that imposes fMRI as a hierarchical soft constraint on EEG for current source estimation (Sato et al., 2004; Yoshioka et al., 2008). This method was selected because it preserves the high temporal resolution of the EEG and the high spatial resolution of the fMRI, and it has been successfully implemented in previous offline BCI studies (Toda et al., 2011; Yoshimura et al., 2012, 2016; Kawase et al., 2017). In the context of this study, a current source can be defined as the average neuronal activation in each 3 × 3 × 3 mm voxel in the brain cortex. The voxels from MRI provide information on the location and orientation of dipoles on the brain cortex, while from the fMRI data the region of interest (area prior) and the relative amplitudes of dipole currents (activity prior) are extracted. Area and activity priors are imposed as a soft constraint to estimate cortical current sources from the EEG data.

In our study, anatomically known areas in the brain contributing to motor planning and execution for ankle movements were obtained from the fMRI analysis. Each participant executed the same ankle movements as experimental tasks during both the fMRI and the EEG experiments, which were carried out separately on different days. Since from the fMRI information we can obtain good anatomical locations, with high resolution, for brain activations related to foot movements, we expect that the estimated current sources are a reasonable representation of the true current sources (group of neurons in each voxel) generated in the brain cortex. After current sources were estimated, we apply a multiclass classifier based on sparse logistic regression (SLR) (Yamashita et al., 2008), for the time series signals of the estimated current sources and EEG sensor signals, to classify ankle flexion and extension with two different force levels (i.e., nine tasks for both legs including a no-motion condition). This method was selected because it is suitable for brain activity data with high-dimensional features, and it has been reported to be more robust in the presence of irrelevant features when compared to other methods such as support vector machine and regularized logistic regression (Yamashita et al., 2008).

Our objective in this research is to estimate cortical current sources from EEG and fMRI recordings, and decode activation patterns in the brain for ankle flexion and extension movements at two force levels in both legs. These motor tasks were selected because of the major role these tasks play in the normal walking cycle, and because the classification of these tasks in healthy participants shows the feasibility to design control strategies for walking aids for patients with walking impairments. Based on the methods described here, classifiers can be created, for example, from motor imagery of ankle movements in patients with spinal cord injury, or from the healthy brain activation related to contralateral ankle or foot movement in patients affected by stroke.

## Materials and methods

### Participants

Eight healthy participants (5 males and 3 females) aged 22–50 years (Mean: 29.67 ± 8.81) participated in this study. Signed informed consents approved by the ethics committee of the National Center of Neurology and Psychiatry (NCNP) and Tokyo Institute of Technology were obtained from each participant prior to each experiment.

### Experimental design

**Figure 1:**
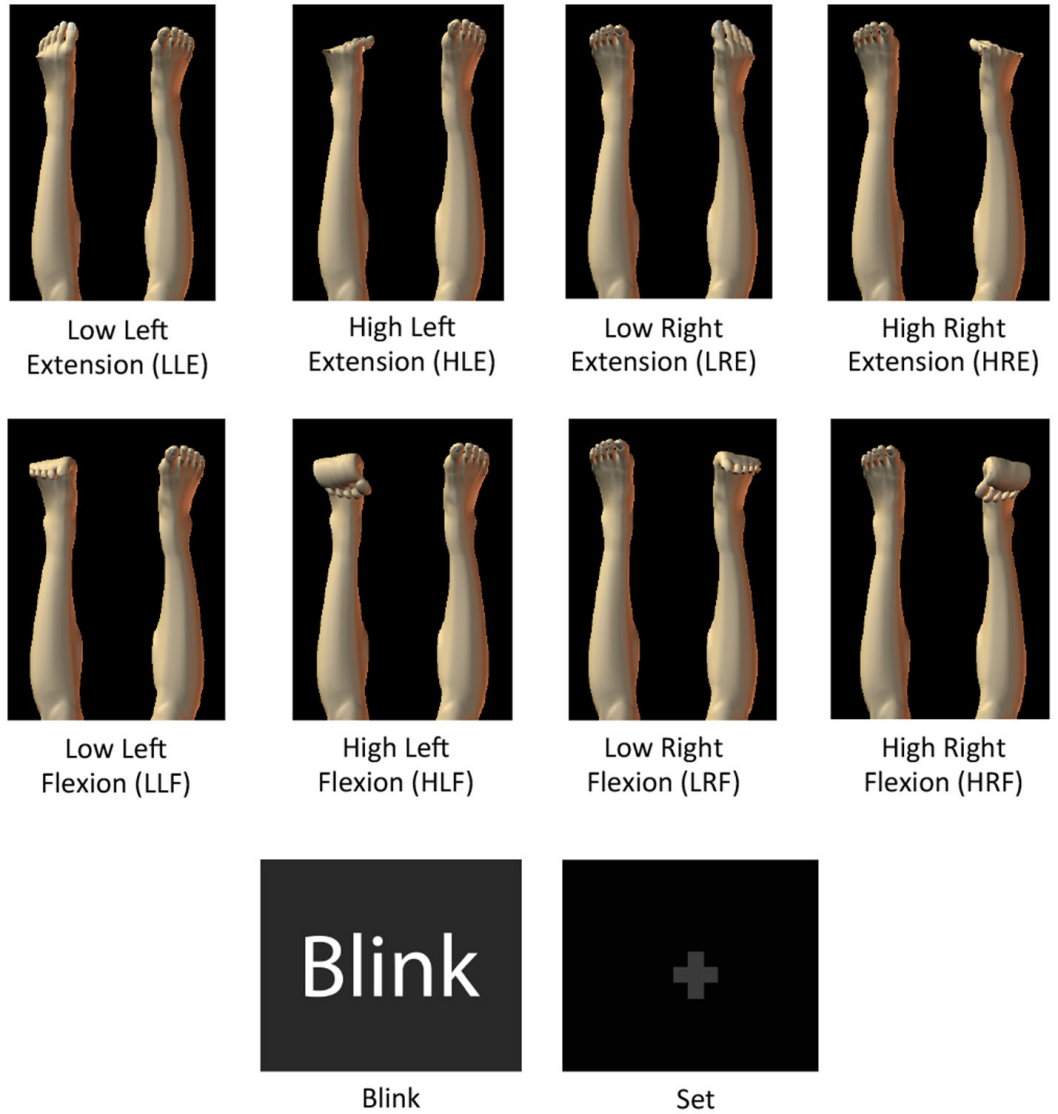
Images with motor task instructions used during the fMRI and the EEG experiments. Blink and fixation crosses were only used for the EEG experiment.

Two types of experiments were conducted in different days: an fMRI and an EEG experiment. The experimental tasks consisted of isometric ankle flexion and extension at high (< ~30% of maximum voluntary contraction level) and low force levels (about half of the high force) in each foot, yielding 8 active task conditions named “High Left Extension” (HLE), “High Left Flexion” (HLF), “High Right Extension” (HRE), “High Right Flexion” (HRF), “Low Left Extension” (LLE), “Low Left Flexion” (LLF), “Low Right Extension” (LRE), “Low Right Flexion” (LRF), and a resting control condition called “Still.” Images showing the motor tasks throughout the experiments were created in Poser 2012 (Smith Micro Software, Inc., California, United States). In both fMRI and EEG experiments the same task pictures were shown, however in the EEG experiment, additional figures indicating the “blinking” and “set” intervals (fixed crosses before each task to reduce eye-movement artifacts in task periods), were included (Figure 1).

### fMRI experiment

The fMRI experiment was conducted to obtain the activated brain areas and the corresponding intensities to the experimental tasks. This information was used as prior for the cortical current sources estimation using the variational Bayesian multimodal encephalography (VBMEG) toolbox for Matlab (Sato et al., 2004).

**Figure 2:**
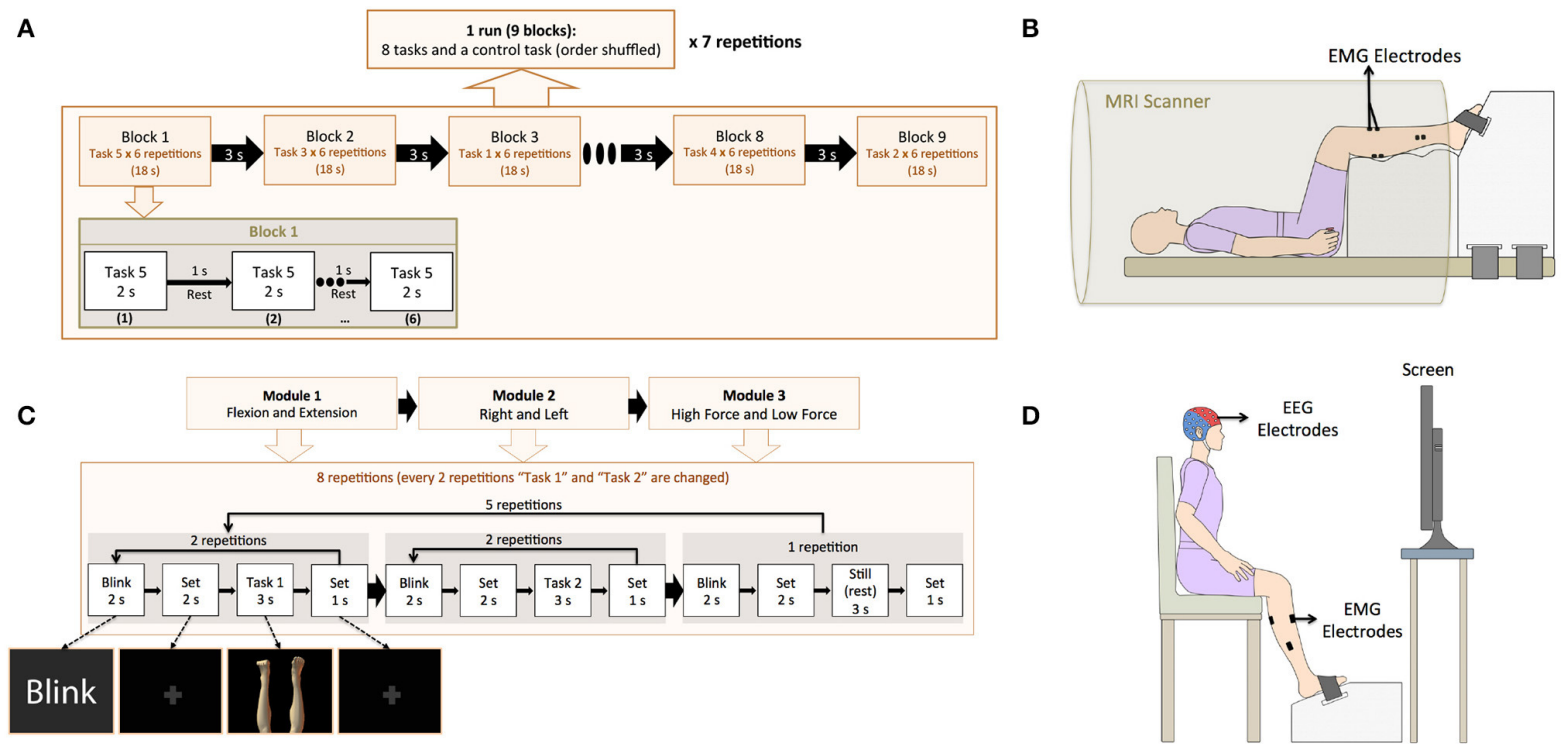
**(A)** Experimental paradigm for the fMRI experiment. The fMRI experiment consisted 7 runs. In each run, 9 blocks were included (8 active tasks and a control task), and each block consisted of one experimental task and a rest interval, both repeated 6 times. **(B)** Experimental setting for the fMRI experiment. EMG electrodes were attached to the participants to confirm task execution and the feet of the participant were fixed to a custom made-platform to reduce head movements inside the scanner. **(C)** Experimental paradigm for the EEG experiment. The EEG experiment consisted of three modules: “Flexion vs. Extension,” “Right vs. Left,” and “High Force vs. Low Force.” In each module, 2 active tasks were repeated 10 times (10 trials) and a still task was repeated 5 times (5 trials). The 2 active tasks were selected based on the module (i.e., HLE and HLF for the “Flexion vs. Extension” module). After 25 trials (10 trials for each of the 2 active tasks and 5 trials for the still task), the active tasks were changed until completing all the experimental tasks. **(D)** Experimental setting for the EEG experiment. A cap with 32 EEG electrodes and 8 EMG electrodes were attached to the participant. A custom-made platform was also used in this experiment to attach the participant's feet during the EEG experiment in order to reduce movement artifacts and allow for isometric contractions.

A block design was used for the fMRI experiment. In total, the experiment consisted of 7 runs with 8 tasks blocks and one still (control) block per run, as detailed in Figure 2A. Each block consisted of one experimental task repeated 6 times during 2 s with 1 s rest (18 s per block). The experimental program was created using Presentation 16.3 (Neurobehavioral Systems, Inc., California, United States).

This experiment was conducted in the National Center of Neurology and Psychiatry (Tokyo, Japan) in a 3 Tesla Verio MRI Scanner (Siemens AG, Munich, Germany). Axial and sagittal scans were acquired for the T1-weighted structural images with a magnetization prepared rapid gradient-echo (MPRAGE). Both images were used during the preprocessing of fMRI; however, the sagittal image was also used to obtain a polygon model of the brain surface for each subject. In total, 48 slices were obtained for the axial images (repetition time = 2 s; echo time = 3.4 ms; flip angle = 8°, field of view = 192 × 192 mm; imaging matrix = 192 × 192; voxel size = 1 × 1 × 1 mm; inversion time = 0.99 ms), and 224 slices were acquired for the sagittal images (repetition time = 2 s; echo time = 3.41 ms; flip angle = 8°; field of view = 256 × 256 mm; imaging matrix = 256 × 256; voxel size = 1 × 1 × 1 mm; inversion time = 0.99 ms). T2^*^-weighted fMRI data was obtained with an echo planar imaging (EPI) with a generalized autocalibrating partial parallel acquisition (GRAPPA) method, recording 116 volumes per session (repetition time = 2 s; echo time = 13 ms; echo train length = 31 ms; flip angle = 90°; field of view = 192 × 192 mm; imaging matrix = 64 × 64; number of slices = 48; voxel size = 3 × 3 × 3 mm).

Electromyography (EMG) of ankle flexors and extensors was recorded during the fMRI experiment with the purpose of confirming task execution. EMG electrodes were attached prior to the fMRI experiment to the Tibialis Anterior (dorsiflexor), the Gastrocnemius (plantarflexor and knee flexor), the Soleus (plantarflexor), and the Extensor Hallucis Brevis (toes extensor) in both legs, and the participant was asked to practice the experimental tasks. EMG data in this experiment was collected with a BrainAmp ExG MR (Brain Products GmbH, Gilching, Germany) using 8 pairs of Ag/AgCl electrodes. Inside the scanner, the feet of the participants were fixed to fMRI compatible custom made platforms (Right Mfg. Co., Ltd, Tokyo, Japan) with detachable Velcro stripes, to allow them to exert isometric force, and to reduce head motions inside the MRI scanner caused by the leg movement tasks (Figure 2B).

### EEG experiment

The EEG program for experiment instruction was created in MATLAB 2013b (The MathWorks, Inc., United States). In this experiment, additional images for blinking and set (fixation crosses indicating the participant to prepare for the task) were included. This experiment was divided into 3 modules: “Flexion vs. Extension,” “Right vs. Left,” and “High Force vs. Low Force,” and participants were asked to take a rest after each module completion. The EEG experiment was designed in this manner attempting to reduce the potential mental fatigue of the participants (Faber et al., 2012; Talukdar and Hazarika, 2017), in consideration of the number of experimental tasks (9 motor tasks with 50 repetitions each), the introduction of task irrelevant images for blink and set (fixation crosses) events, and the condition of exerting isometric forces. Four runs composed each module and each run was repeated twice. According to the current module, each run combined two tasks and a still task, that is, in the “Flexion vs. Extension” module the tasks “LLF and LLE,” “HLF and HLE,” “LRF and LRE,” and “HRF and HRE” and still, were included (Figure 2C).

In preparation for the EEG experiment, the participants were seated inside a soundproof room (AMC-3515, O'HARA & Co., Ltd.) with a 24 inches monitor to show the experiment directions. The participant feet were fixed to the platform and instructed to practice ankle flexion and extension at high and low force levels to learn to restrain co-contraction of flexor and extensor muscles (Figure 2D).

EEG signals were acquired with a sampling rate of 256 Hz with the ActiveTwo system and the ActiView software (BIOSEMI, Amsterdam, Netherlands), using 32 Ag/AgCl active electrodes placed accordingly to the 10–20 international system layout. Two additional electrodes were placed on both earlobes and its average was used as a reference. To place the EEG and the reference electrodes, the head cap gaps were filled with highly conductive gel and the earlobes were cleaned with 70% ethanol. EEG electrodes positions were recorded with a Polaris Spectra (Northern Digital Inc., Waterloo, Canada). Measurements were done in the order of nasion, right pre-auricular, left pre-auricular, and EEG electrodes according to the BIOSEMI electrodes labels.

To confirm task execution in the EEG experiment, EMG signals were recorded a sampling rate of 2,000 Hz with a Bagnoli™ Desktop EMG System (Delsys, United States) using 8 single differential electrodes on the same muscles as the fMRI experiment. EMG signal conditioning and digitalization was done with a NI-USB 6259 BNC (National Instruments, Canada).

### fMRI data preprocessing

fMRI data was processed using SPM8 (Wellcome Department of Cognitive Neurology, UK; http://www.fil.ion.ucl.ac.uk/spm), for individual and group (second-level) analyses. In preparation for the analyses, the first five volumes of the EPI images were discarded for stabilization of the magnetization, and the last 10 volumes were discarded to avoid their use as a baseline, therefore, from the original 116 volumes, 101 volumes were used for the analyses. For the individual analyses T1-weighted axial and sagittal images were bias corrected and segmented into gray matter, white matter and cerebrospinal fluid. EPIs were corrected for differences in image acquisition time, and realigned to the mean EPI image. To register all images, the T1-weighted axial image was co-registered to the T1-weighted sagittal image and then the EPIs were co-registered to the T1-weighted axial image. It is worth mentioning that this co-registration method is not standard for fMRI analysis, and it is used only for current source estimation purpose in VBMEG. After images registration, all images were normalized to the Montreal Neurological Institute (MNI) coordinates, and smoothed with a full-width spatial Gaussian kernel of 8 mm at half maximum.

Statistical analyses were performed with a general linear model (GLM). Boxcar functions were used to model the nine periods corresponding to the nine blocks. Each execution period consisted of one block of 18 and 3 s of rest interval. These functions were then convolved with the hemodynamic response function to obtain parameters describing the blood oxygen level-dependent (BOLD) response at each stimulus presentation (task image). Finally, model parameters were estimated and statistical parametric maps were created for each participant. Using the contrasts obtained from individual analyses, a second-level (group) analysis was done with a full factorial design to extract parametric maps common for all participants. Three factors were used for the design: “Factor 1: left leg and right leg,” “Factor 2: flexion and extension,” and “Factor 3: high force and low force.” T-contrasts were obtained for each of the following conditions: (all left leg tasks) and (all right leg tasks) with *p* < 0.01 (uncorrected for multiple comparisons). Statistical parametric maps from the group analysis were masked with the anatomical atlas of Brodmann areas 4 (primary motor cortex), 6 (premotor cortex and supplementary motor area), and 3,2,1 (primary somatosensory cortex) using the WFU PickAtlas (Radiology Informatics and Imaging Laboratory, USA; http://fmri.wfubmc.edu/software/pickatlas) tool for SPM to build the area and activity priors for the purpose of cortical current sources estimation in VBMEG. The two contrasts obtained were inversely normalized into individual participant's space and merged into one activity prior and one area prior. These priors were named Group-Con.

### EEG data preprocessing

EEG data recorded in BDF format from BIOSEMI was converted into Matlab format with EEGlab (Delorme and Makeig, 2004, https://sccn.ucsd.edu/wiki/EEGLAB), band-pass filtered from 0.5 to 40 Hz, downsampled to 200 Hz, and epoched in intervals of −0.5 s pre onset and 3 s post onset, in reference to the stimulus presentation time. This pre-processed EEG was further downsampled to 30 Hz and epoched from 0 s (onset) to 1.5 s, and these epochs of 1.5 s were used as features for the sparse logistic regression classifier, to obtain the EEG sensor signals that contributed to each of the nine experimental tasks.

### Current source estimation with VBMEG

VBMEG is a type of distributed source method in which the MRI information provides information about the positions and orientations of dipoles, and the fMRI provides information about a region of interest and the relative amplitude of the current in each dipole (Yoshioka et al., 2008).

In conventional current estimation methods where the fMRI is also used as a prior, the fMRI information is imposed directly as the prior current variance in each dipole, and therefore the current amplitude has a low influence when the prior variance is too large or too small. In VBMEG, the prior distribution of the variance is considered a random parameter with gamma distribution, and the fMRI information is imposed on the variance distribution, rather than as the variance itself, using two hyperparameters: a variance magnification parameter (μ_0_), controlling the current amplitude for a given fMRI activation, and a confidence parameter (γ_0_), controlling the width of the prior distribution. This hierarchical prior provides a soft constraint on the current amplitude. A spatial smoothness constraint with a Gaussian profile with a full width at half maximum (FWHM) of 6 mm, was incorporated in the estimation. This smoothness constraint considers that neurons within a few millimeters radius tend to fire simultaneously (Sato et al., 2004; Yoshioka et al., 2008; Toda et al., 2011; Yoshimura et al., 2012).

Because of the hierarchical prior, the estimation of the inverse filter becomes a non-linear problem that cannot be solved analytically, therefore the approximate posterior distribution is calculated by using a Variational Bayesian (VB) method, in which the current and the variance from the observed EEG data and the prior variance information given by the fMRI data are alternately estimated. The inverse filter is calculated using the estimated covariance matrix in the previous iteration (Attias, 1999; Sato, 2001). Once the inverse filter has been calculated, the current estimation becomes al linear problem.

Current sources were estimated in VBMEG following the standard procedures established in the toolbox documentation. The following steps and parameters were used to estimate cortical currents in VBMEG: firstly, a cortical surface model and a three-layer head model for each participant were extracted from the un-normalized bias-corrected T1-weighted sagittal image from the SPM analysis. The cortical surface model was created as a polygon model using FreeSurfer (Martinos Center Software, https://surfer.nmr.mgh.harvard.edu/). The cortical surface model has single-current dipoles equidistantly distributed on and perpendicular to the cortical surface, and the three-layer model has the boundary information for skull, scalp, and cerebrospinal fluid. VBMEG imports the cortical model to map the estimated current dipoles, and uses the three-layer model to create a head model for improving the accuracy in the leadfield (i.e., forward model) calculation.

Secondly, the leadfield matrix is calculated from the cortical surface model, the head model and the EEG sensor positions. Thirdly, the variance of the electrical current from EEG is estimated in the time range from −0.5 to 3 s with a baseline from −0.5 to 0 s, and the fMRI information is imposed on the prior distribution of the current variance using the hyperparameters μ_0_ and γ_0_. High values for both hyperparameters indicate that the brain activity was the same during both fMRI and EEG experiments. Considering the experiments were carried out in different days, and based on the previous work (Yoshimura et al., 2016), the hyperparameters values for the Group-Con area and activity priors were set as μ_0_ = 10 and γ_0_ = 1.

To estimate the inverse filters, the whole epoch of EEG data (−0.5 to 3 s) was used for the analysis, being divided into 14 windows of 0.5 s of length with 0.25 s of overlap. This setting calculated an inverse filter for each time window corresponding to each epoch and trial. These current sources were estimated for the area and activity priors determined by Group-Con. The mean number of current dipoles estimated for each participant was of 188 ± 7.07. The time series of estimated current sources were further downsampled to 30 Hz and epoched from 0 s (onset) to 1.5 s, and these epochs of 1.5 s were used as features for the sparse logistic regression classifier, to obtain which current sources contributed most to each of the nine experimental tasks.

### Multi-class classification with sparse logistic regression

Logistic regression (LR) is a well-known classifier originally developed in statistics. SLR is a Bayesian extension of LR in which a sparseness prior is imposed on LR (http://www.cns.atr.jp/~oyamashi/SLR_WEB/Readme201102.pdf). The SLR method combines the LR with the automatic relevance determination (ARD), to simultaneously perform feature selection and training of the model for classification. The ARD prunes irrelevant features by automatically setting their associated weights to zero, leading to a sparse weight vector for classification. This allows the SLR to train high-dimensional classifiers without the need of advanced feature dimension reduction, and to avoid overfitting to some extent. SLR was applied in this research using the SLR Toolbox v.1.2.1 (ATR Computational Neuroscience laboratories in Kyoto, Japan; http://www.cns.atr.jp/~oyamashi/SLR_WEB.html).

Multiclass classifications were done for the epochs of 1.5 s of estimated current sources and EEG sensor signals, using a leave-one-out (LOO) method for both current sources and pre-processed EEG data. The features for classification were obtained as described in section EEG Data Preprocessing for EEG and section Current Source Estimation with VBMEG for current sources. Mean classification accuracies from current sources, EEG and random labeled data were compared using non-parametric permutation tests (Nichols and Holmes, 2002). To evaluate the contribution of current sources and EEG sensors to each task classification, the corresponding weight values were normalized by the maximum of each trial and averaged across time points and trials. Finally, net weight values of the current sources vertices selected in each area in our ROI, and weights in each EEG sensor were averaged across participants. Location of contributing selected current sources was determined using the Anatomy toolbox v1.8 [Institute of Neuroscience and Medicine (INM-1), Germany], using the Montreal Neurological Institute (MNI) coordinates obtained from SPM.

## Results

### Classification results

**Figure 3:**
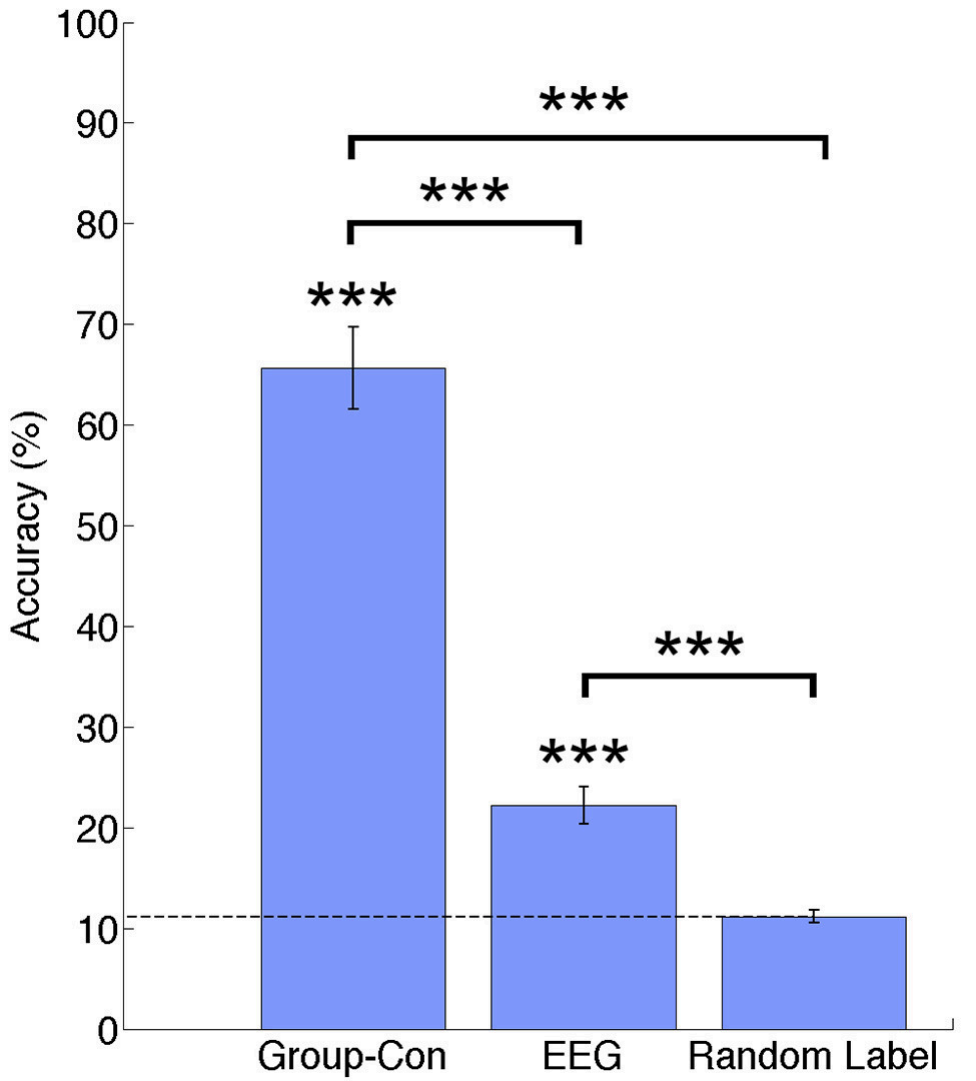
Classification accuracies across participants for current sources estimated using priors from and fMRI group analysis (Group-Con), pre-processed EEG signals (EEG), and a random label classification for current sources. ^***^p < 0.001.

In Figure 3 percentage of correct classifications in the test data from current sources, EEG and random label are shown. The classification accuracy from current sources was significantly higher than chance level (Current sources: 65.64% ±4.11; *p* = 1.19e−04), and EEG sensor signals (EEG: 22.19%; *p* = 1.19e-04). All *p*-values were obtained from permutation tests and corrected for multiple comparisons using a false discovery rate of 0.05. There was no significant difference between the random label classification and the chance level.

Tables 1, 2 show the confusion matrix for the current sources classification and pre-processed EEG classification for all trials averaged across participants.

**Table 1:** Confusion matrix for current sources classification averaged across participants (50 test trials per class).

	**Current sources classification accuracies (%)**								
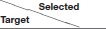	**HLE**	**HLF**	**HRE**	**HRF**	**LLE**	**LLF**	**LRE**	**LRF**	**Control**
HLE	31.63	2.63	2.25	1.25	2.25	2.63	2.00	1.63	3.75
HLF	1.00	35.50	1.25	1.88	2.50	3.25	1.38	1.00	2.25
HRE	2.38	1.38	35.38	1.38	1.38	2.00	3.25	1.00	1.88
HRF	1.00	2.25	1.63	34.63	1.00	2.38	1.88	3.13	2.13
LLE	1.50	2.75	2.00	1.38	32.75	2.75	2.13	1.88	2.88
LLF	1.88	3.38	1.25	2.38	3.25	29.75	2.13	2.00	4.00
LRE	1.25	1.75	2.13	2.63	2.63	3.13	29.75	2.38	4.38
LRF	2.00	1.50	1.00	2.25	1.13	3.38	2.13	34.25	2.38
Control	1.88	1.38	1.25	1.25	3.25	4.38	2.75	2.13	31.75
Mean accuracy per class (%)	63.26 ± 8.28	71 ± 8.90	70.76 ± 6.26	69.26 ± 6.80	65.5 ± 8.40	59.50 ± 11.99	59.50 ± 6.58	68.5 ± 6.59	63.50 ± 7.11
Mean accuracy (%)	65.64 ± 4.11								

**Table 2:** Confusion matrix for EEG sensor signals classification averaged across participants (50 test trials per class).

	**EEG sensor signals classification accuracies (%)**								
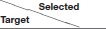	**HLE**	**HLF**	**HRE**	**HRF**	**LLE**	**LLF**	**LRE**	**LRF**	**Control**
HLE	9.00	6.50	5.75	4.75	6.00	6.38	4.13	3.88	3.63
HLF	7.13	9.13	4.13	4.88	5.75	7.25	3.13	3.38	5.25
HRE	4.00	4.25	10.75	8.25	3.38	3.75	6.63	6.00	3.00
HRF	4.88	4.88	6.75	11.13	3.25	3.25	4.75	6.75	4.38
LLE	5.88	6.00	4.38	3.00	10.63	7.50	5.13	3.25	4.25
LLF	7.13	6.25	4.50	2.50	6.00	9.50	4.38	3.88	5.88
LRE	5.25	3.50	6.50	5.38	4.63	3.88	10.63	6.25	4.00
LRF	4.00	3.00	4.75	7.75	3.63	3.88	6.38	12.63	4.00
Control	3.13	5.25	2.25	3.50	4.38	5.38	4.38	5.25	16.50
Mean accuracy per class (%)	18 ± 3.25	18.25 ± 2.70	21.5 ± 2.76	22.25 ± 3.64	21.25 ± 3.81	19 ± 2.45	21.25 ± 3.85	25.25 ± 4.41	33 ± 8.52
Mean accuracy (%)	22.19 ± 1.85								

### Localization of contributing features

**Figure 4:**
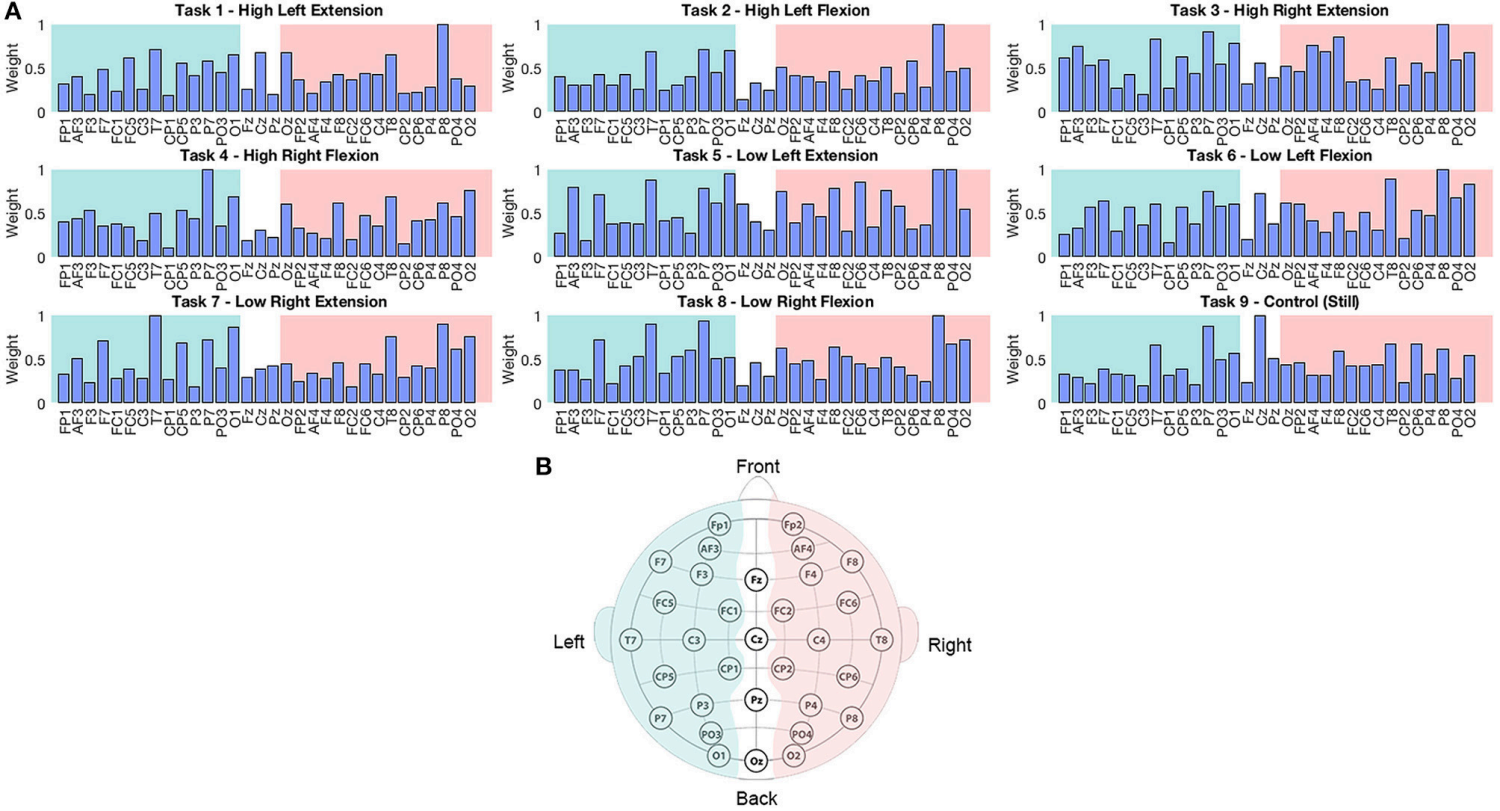
**(A)** Normalized weights obtained for each task in EEG classification. Bars located on the green area correspond to the sensors located in the left hemisphere, bars in the white area correspond to the midline, and electrodes in the pink area correspond to the right hemisphere. **(B)** Location of 32 EEG electrodes over the scalp using the 10–20 extended system.

**Figure 5:**
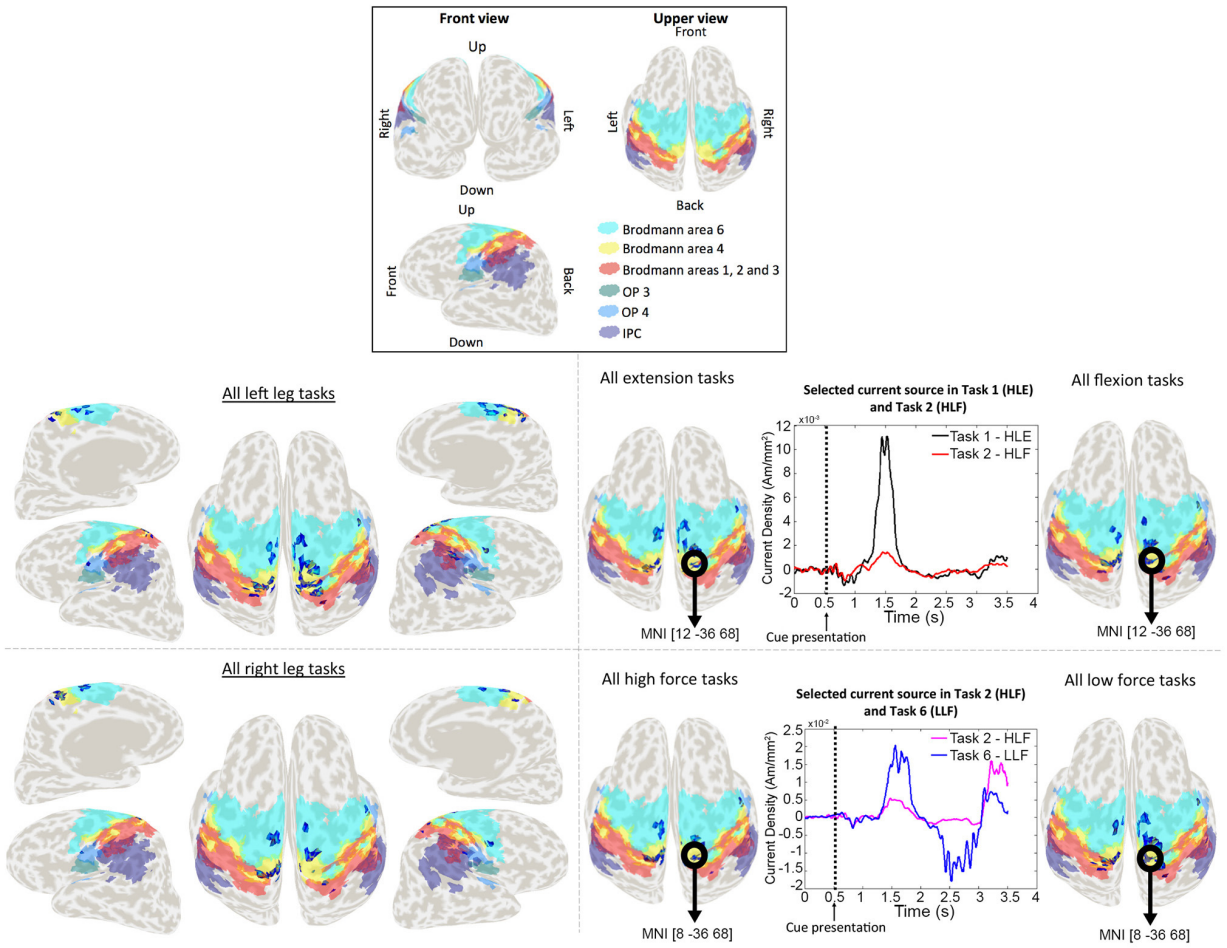
Activation patterns for a representative participant. Colored areas show Brodmann areas 1, 2, 3, 4, 6 OP3, OP4, and the IPC. Lower left panel shows the merged activation patterns for all left and right leg tasks (all right leg tasks: HRE + HRF + LRE + LRF; all left leg tasks: HLE + HLF + LLE + LLF), and lower right panel shows the activation patterns for all extension tasks, all flexion tasks, all high force tasks and all low force tasks (all flexion tasks: HRF + HLF + LRF + LLF; all extension tasks: HRE + HLE + LRE + LLE; all high force tasks: HRF + HLF + HRE + HLE; all high force tasks: LRF + LLF + LRE + LLE). Time series represent the temporal patterns for the current source vertex in bold black circles. These signals correspond to a vertex selected by the classifier as relevant for more than one task. The vertex located in MNI [12, −36, 68], was selected by the classifier for both HLE and HLF task, and the vertex located in MNI [8, −36, 68] was selected for both HLF and LLF tasks.

Weight analyses were performed for EEG and current sources classification results. For EEG, weights were averaged and normalized across trials and participants in each EEG sensor (Figure 4). For current sources, total weights across dipoles located in the same Brodmann area were averaged across trials and participants, and normalized per task. Current sources were obtained across Brodmann areas 1, 2, 3, 4, 6, the inferior parietal cortex (IPC) and the lateral operculum (OP3 and OP4). Finally, activation patterns were obtained for each participant showing the current sources distribution in each task (Figure 5).

## Discussion

This research presented a multi-class classification analysis of ankle motor tasks using non-invasive brain recorded signals from EEG and fMRI. As a result, we could successfully identify activation patterns for flexion and extension tasks at two different force levels in both feet. Due to the difficulty of measuring brain activity during walk related motor tasks inside the fMRI scanner, our approach focuses on the classification of ankle flexion and extension tasks that can provide an insight of control for a real time walking BCI. We obtained accuracies of 65.64% for the classification of estimated current sources, and of 22.19% for the classification of EEG sensor signals above chance level (11.11%) and no significant difference was found among classes across participants showing no disproportion of true positives.

The high classification accuracy could be attributed to the combination of VBMEG and SLR. From VBMEG, many redundant (but not identical) time series patterns of current sources are estimated and this output could give SLR many candidates for features selection from the sparse regularization point of view (Donoho, 2006). As a result, many similar time series patterns remain for classification producing high classification accuracies.

Additionally, the high-resolution fMRI prior may have also contributed to the high classification accuracy despite the use of 32 EEG channels. While a low resolution prior and a low number of electrodes affects the quality of the current sources estimated in VBMEG, and consequently the classifications results, the high spatial resolution area prior may be more important for proper current source estimation in VBMEG, than the number of EEG electrodes: a study performed by Aihara et al. (2012) showed that the detection accuracy of current sources from simulated EEG data and spatial priors at different resolutions, was largely affected by the spatial resolution of the prior than by the number of EEG sensors. Similar results were obtained with the experimental data, using 19, 31, and 64 EEG channels, and fMRI and near-infrared spectroscopy (NIRS) as priors. The fMRI prior and 64 EEG channels estimation was used as a reference. In both simulated and real data cases, the use of 19 EEG channels along with the lowest resolution prior (NIRS in the case of real data), outperformed the use of 64 EEG channels without any prior information for current source estimation.

Currently it is not possible to isolate or identify individual muscle activity from EEG recordings, however a previous study by Yoshimura et al. (2012) succeeded in reconstructing individual muscle activities (flexor carpi radialis and extensor carpi radialis brevis) from cortical current sources estimated from 32 EEG electrodes using VBMEG and a sparse regression method, in a five task experimental paradigm of wrist flexion and extension at high and low forces. These results further support the high performance of the current source estimation method with VBMEG. As for this study, we have partially succeeded in the reconstruction of ankle flexor (tibialis anterior) and extensor (soleus) muscles activities using the methods described in the paper.

As for the classification technique, we selected SLR as a classifier because it has a better classification performance in the presence of irrelevant features, when compared to more popular methods such as support vector machine (SVM) and regularized logistic regression (RLR; Yamashita et al., 2008). Additionally, in the multiclass classification each class has its own set of parameters, which allowed obtaining activation patterns of current sources that are specific to each of the experimental tasks.

### Location of features contributive to task execution

The weights assigned by the classifier to EEG electrodes did not offer relevant information about the execution of the motor tasks of interest, however this result was reasonable considering the low classification accuracy of these signals. Higher weights tended to be assigned to electrodes placed over temporal and parietal lobes (P7, P8, T7, and T8; Figure 4). This outcome could be related to artifacts caused by eye movement or EMG artifacts during the isometric task execution.

In the case of current sources classification, we obtained different activation patterns distributed over known brain areas involved in motor planning and execution. Vertices that were selected by the classifier as relevant for more than one task, had different signal patterns to which we attribute the higher performance classification when compared to EEG sensor signals classification. These activation patterns are shown in Figure 5 for a representative participant. For conciseness, in Figure 5 single task patterns were merged into (1) left leg versus right leg tasks, (2) flexion versus extension tasks, and (3) high force versus low force tasks.

The group analysis showed current sources widely distributed along our ROI of Brodmann areas 1, 2, 3 (primary somatosensory cortex), 4 (primary motor cortex), and 6 (premotor cortex and supplementary motor area), however it also showed activations in the IPC and the OP. The activations in these areas adjacent to Brodmann areas 1, 2, 3, 4, and 6 were reasonable considering the co-registration and normalization methods of fMRI and MRI data are not optimal processes, and the inverse normalization is as good as the initial normalization to the standard brain for each participant.

Current sources located in the ipsilateral side of the brain (resting leg) were observed throughout participants. While these activations were expected by the use of a single fMRI prior and co-contractions during task execution, these activations are also reasonable considering that unilateral limb movements are not exclusive of the contralateral brain and also show activations on the ipsilateral brain (Chiou et al., 2013).

### Brain areas contributive to task execution

Somatosensory information from Brodmann areas 1, 2, and 3 is used in motor control: area 3 receives information relevant for proprioception and skin touch, which is processed with areas 1 and 2 (Amaral, 2013). The primary motor cortex and the primary somatosensory cortex have shown prominent activations during walking experiments (la Fougère et al., 2010), which are strongly related to the goal of this study. Brodmann area 6 is involved in preparing and organizing voluntary movements (Cunnington et al., 1996; Sira and Mateer, 2014), and the highest activation in this area across participants may be related to the complexity of the task in which not only flexion and extension movements needed to be prepared for both lower limbs, but also force modulation was required. Activation in the IPC was observed in the rostral and middle areas. The rostral areas are functionally connected to motor, premotor, and somatosensory areas, thus this activation may be related to sensorimotor integration of motor task observation and task execution (Caspers et al., 2011), while middle IPC is involved visually guided attention, and it may have aroused as a result of the fixation crosses used during the EEG experiment (Caspers et al., 2013). The operculum (OP3 and OP4) is part of the secondary somatosensory cortex (SII). Studies in the role of SII in sensory-motor integration (Inoue et al., 2002) and its somatotopic map, have reported activations in SII as a result of mechanical plantar stimulation (to produce a gait-like somatosensory inflow; Labriffe et al., 2017) and somatosensory stimulation in the legs, trunk, hands and head (Disbrow et al., 2000; Eickhoff et al., 2007).

We also assessed the performance of the method in classifying tasks from each brain hemisphere (each leg separately), as a future application for patients with stroke. We created area and activity priors using the t-contrasts from only left leg tasks and only right leg tasks, and masked them with the previously described ROI. Priors in the left brain only were used for right leg tasks classification, and priors in the right brain only were used for the left leg tasks classification, resulting in 5-class classifications (4 active tasks per leg and a control task) with a chance level of 20% for each classification. Accuracies obtained from the current sources classification was significantly higher than EEG and chance level in each left leg (current sources: 70.55% ± 5.31; EEG: 31.40 ± 2.69; *p* = 1.19e−04 corrected for multiple comparisons) and right leg (current sources: 73.55 ± 2.82; EEG: 32.10 ± 3.07; *p* = 1.19e−04 corrected for multiple comparisons).

### Advantage of using group fMRI priors for current source estimation

Various analyses using anatomical priors and fMRI priors obtained from individual analyses were also conducted in this study, obtaining current source classification accuracies similar to the group analysis (9-class mean classification accuracy in individual space: 69.25% ± 2.61). While the number of participants needs to be increased in order to draw more robust conclusions specifically for an fMRI second-level analysis, the purpose of using the group prior is to show that the methods described here are applicable using only structural MRI data, and therefore can benefit patients unable to use the MRI scanner for long sessions.

### BCIs application in rehabilitation

Invasive BCI recordings in non-human primates have successfully been used for decoding kinematic and physiological activities (EMG), during forward and backward walking on a treadmill. In humans, the use of this method, along with exoskeletons or FES systems, could make it possible to create walking strategies that facilitate spinal cord plasticity to help recovering locomotion automatisms (Fitzsimmons et al., 2009). As for non-invasive techniques, the use of different strategies such as virtual reality, lower limb actuators, exoskeletons, etc., in patients with paraplegia, have already shown significant improvement in functional cortical plasticity in S1 and M1 areas (Donati et al., 2016). In this sense, the methods described here, along with high density EEG, might allow also for the prediction of kinematic variables in patients with limited mobility, aiming to design a control strategy where the patient has more control over the system than a robotic or neurostimulation solution.

In this study we have further assessed the potential of the VBMEG and SLR methods to design BCIs to control assistive and rehabilitation devices for the restoration of walking in patients with motor impairments. The highly enhanced spatial distribution of current sources over the brain cortex has the potential to identify changes in cortical plasticity in patients with stroke, to design interfaces that can identify vertices in the healthy brain relevant to the affected limb control, or to design BCIs for patients with spinal cord injury from current source estimation from motor imagery.

### Challenges toward the development of a real-time BCI

In order to design a reliable real time BCI, the accuracy of the system needs to be increased and a generalized classifier needs to be developed. In our experiment, blinking sessions were performed independently of task execution sessions, therefore online rejection of blinking artifacts should be implemented for the online application. One of the most challenging goals in this project is to develop a generalized classifier able to achieve high accuracies with all participants, due to, among other factors, the non-stationary nature of brain signals, parameter tuning and training of samples, internal artifacts, etc. Finally, a limitation in this study is the small sample of participants used and therefore it is necessary to increase the sample and extend these conclusions to a larger population.

## Conclusion

In this study we have classified ankle flexion and extension movements at different force levels in healthy participants, using non-invasive brain activity recording methods. The technique applied in this research is applicable to real-time BCIs since the filters estimation can be done offline. Also, the nature of the recordings allows for this technique to be applied to a larger population of patients with motor impairments since it does not require surgery. Finally, different combinations of area and activity priors from fMRI can be applied, and therefore specific brain areas may be used to generate control strategies in patients with stroke or spinal cord injury.

## Ethics statement

This study was carried out in accordance with the recommendations of the guidelines A14036 from Tokyo Institute of Technology and A2014-020 from the National Center of Neurology and Psychiatry, with written informed consent from all subjects. All subjects gave written informed consent in accordance with the Declaration of Helsinki. The protocol was approved by the ethics committees of the National Center of Neurology and Psychiatry and Tokyo Institute of Technology.

## Author contributions

AM: designed and performed the EEG experiment, analyzed the fMRI and EEG data, reviewed literature and drafted the manuscript; RH, YO, and NY: designed and performed the fMRI experiment and analyzed data; KK, TN, and HK: performed the fMRI experiment; TH, YK, and NY: supervised the experiments and the research.

### Conflict of interest statement

The authors declare that the research was conducted in the absence of any commercial or financial relationships that could be construed as a potential conflict of interest. The reviewer FF and handling Editor declared their shared affiliation.
